# THE ROLE OF THIRD PLACES IN REDUCING LONELINESS AMONG CAREGIVING SPOUSES

**DOI:** 10.1093/geroni/igac059.2111

**Published:** 2022-12-20

**Authors:** Yeon Jin Choi, Jennifer Ailshire

**Affiliations:** University of Southern California, Los Angeles, California, United States; University of Southern California, Los Angeles, California, United States

## Abstract

Loneliness is a public health concern that is associated with poor mental and physical health. Caregiving spouses of community-dwelling older adults often have high levels of caregiving burden, which make them more vulnerable to social isolation and loneliness. There has been a growing interest in third places as mediums for social interaction. Research on third places shows a positive association of greater access to third places with social networks and social health. However, this has not been tested in the context of caregiving. In this paper, we examined the role of various types of third places in reducing loneliness among caregiving spouses. We used the 2006-2016 Health and Retirement Study and the National Neighborhood Data Archive to examine the relationship between the availability of third places per square mile and loneliness. Third places include food outlets (e.g. grocery stores), eating and drinking places (e.g., restaurants, coffee shops), commercial establishments (e.g., department stores), entertainment organizations (e.g., museum), exercise facilities (e.g., fitness), religious organizations (e.g., churches), civic and social organizations (e.g., social clubs), personal care services (e.g., barbershops, beauty salons), and social services for older adults (e.g., senior centers). We found that caregiving spouses living in neighborhoods with greater availability of third places had lower levels of loneliness. We also found gender differences in the association. For instance, greater availability of eating and drinking places was associated with loneliness only among females. Increasing access to places that provide opportunities for social interactions may prevent social isolation and reduce loneliness among caregiving spouses.

